# Self-Exfoliated Guanidinium Covalent Organic Nanosheets as High-Capacity Curcumin Carrier

**DOI:** 10.3390/biomimetics9110709

**Published:** 2024-11-19

**Authors:** Archita Sharma, Dhavan Sharma, Hengyu Lin, Hongcai (Joe) Zhou, Feng Zhao

**Affiliations:** 1 Department of Biomedical Engineering, Texas A&M University, College Station, TX 77843, USA; sharma_archita09@tamu.edu (A.S.); ddsharma@tamu.edu (D.S.); 2 Department of Chemistry, Texas A&M University, College Station, TX 77843, USA; kingsleyinpku@tamu.edu (H.L.); zhou@chem.tamu.edu (H.Z.)

**Keywords:** guanidinium covalent organic nanosheets, curcumin, bioavailability, drug carrier, self-exfoliation

## Abstract

Drug administration is commonly used to treat chronic wounds but faces challenges such as poor bioavailability, instability, and uncontrollable release. Existing drug delivery platforms are limited by chemical instability, poor functionality, complex synthesis, and toxic by-products. Presently, research efforts are focused on developing novel drug carriers to enhance drug efficacy. Guanidinium Covalent Organic Nanosheets (gCONs) offer promising alternatives due to their high porosity, surface area, loading capacity, and ability to provide controlled, sustained, and target-specific drug delivery. Herein, we successfully synthesized self-exfoliated gCONs using a Schiff base condensation reaction and embedded curcumin (CUR), a polyphenolic pleiotropic drug with antioxidant and anti-inflammatory properties, via the wet impregnation method. The BET porosimeter exhibited the filling of gCON pores with CUR. Morphological investigations revealed the formation of sheet-like structures in gCON. Culturing human dermal fibroblasts (hDFs) on gCON demonstrated cytocompatibility even at a concentration as high as 1000 µg/mL. Drug release studies demonstrated a controlled and sustained release of CUR over an extended period of 5 days, facilitated by the high loading capacity of gCON. Furthermore, the inherent antioxidant and anti-inflammatory properties of CUR were preserved after loading into the gCON, underscoring the potential of CUR-loaded gCON formulation for effective therapeutic applications. Conclusively, this study provides fundamental information relevant to the performance of gCONs as a drug delivery system and the synergistic effect of CUR and CONs addressing issues like drug bioavailability and instability.

## 1. Introduction

Worldwide, millions suffer from acute and chronic wounds annually [1], with standard treatments like surgical debridement and wound dressings, while novel therapies such as skin substitutes, stem cell therapies, and advanced technologies like 3D bioprinting show potential but face challenges like high costs, limited effectiveness, and slow wound closure rates [2,3]. Drug administration is a well-recognized and straightforward approach to treating chronic wounds. The primary goal of a drug delivery system (DDS) is to extend, confine, and target the drug specifically to the damaged area/wound bed, ensuring a protected interaction releasing a drug at the optimal time, in the precise concentration, and at the intended site [4]. The side effects of the drugs include a limited half-life of the biological factors or the dynamic microenvironment of the wound bed, necessitating the use of DDS to deliver the active factors in the proper dosage, targeting the appropriate location [5]. DDS often employs metallic, organic, inorganic, and polymeric nanostructures to accomplish target-specific delivery. However, these conventional systems face numerous limitations as drug delivery carriers, like toxicity, limited loading capacity, premature drug release, instability, and poor therapeutic outcomes [4]. An effective DDS should overcome these challenges by demonstrating high drug loading capacity, controlled and sustained release, and biocompatibility [6]. 

Numerous drugs such as baicalin [7], silver [8], amphotericin B [9], thrombin [10], and norfloxacin [11] have been used in the DDS for wound treatment and regeneration of the skin. Natural bioactive compounds like phenols have gained considerable attention for facilitating wound healing [12]. Curcumin (CUR), a polyphenolic pleiotropic drug derived from *Curcuma longa*, enhances the healing process across various healing stages, such as inflammation, maturation, and proliferation by suppressing the NF-κB signaling pathway, which is responsible for prolonged inflammation in chronic wounds [13]. The healing potential of CUR is attributed to its antioxidant, anti-inflammatory, and antibacterial properties [14,15]. The antioxidant property is the capacity to neutralize or remove oxygen-free radicals that are formed in excess due to environmental abnormalities. Increased oxidative stress levels have dire consequences, which ultimately result in poor mitochondrial function and, thus, cell death. CUR is a potent scavenger of reactive oxygen species (ROS) and reactive nitrogen species (RNS), and chelates heavy metals, thus, helping to reduce oxidative stress [16]. It also has the potential to block the activity of free-radical activated transcription factor-NF-κB and the production of cytokines from activated macrophages, which helps in regulating the inflammatory process [17]. It is recognized as a naturally occurring, powerful scavenger of numerous free radicals, such as nitric oxide (NO), a short-lived free radical that is generated endogenously, and significantly influences the inflammatory wound-healing process. It is a pro-inflammatory biomarker generated by macrophages. It decreases the amount of nitrite produced during the reaction between oxygen and NO [18]. However, direct curcumin administration suffers inherent restrictions such as poor bioavailability, instability in an aqueous environment, burst, and uncontrollable drug release [19].

Despite the availability of numerous DDS like nanoparticles, hydrogels, and liposomes, these systems often lack chemical instability, poor functional properties, cumbersome synthesis procedures, limited loading capacities, and poor degradability, all of which negatively impact drug delivery performance [6]. Another promising alternative to traditional DDS is metal–organic frameworks (MOFs). However, concerns have arisen regarding their potential toxicity, primarily because of the presence of metal coordination bonds within their structure and chemical instability [20]. Addressing these challenges is pivotal for advancing research and improving DDS. Presently, research efforts are focused on developing novel drug delivery carriers to overcome such limitations to enhance drug efficacy [21]. Of the numerous drug delivery carriers reported, 2D Covalent Organic Nanosheets (CONs) are novel well-ordered porous crystalline polymers with promising potential as drug delivery carriers [22,23]. The backbone of CONs is made up of light elements, like boron (B), carbon (C), nitrogen (N), oxygen (O), and silicon (Si), which are linked via strong covalent bonds defined as the concept of reticular chemistry. This distinctive characteristic provides CONs with better compatibility, structural versatility, greater stability, and functional adaptability as compared to conventional DDS [24,25]. CONs offer diverse benefits, including structural rigidity, robust framework, high drug-loading capacity due to intrinsic porosity and high surface area, and less steric hindrance. Their controlled drug release profile, with the least burst effects, makes them potential candidates for drug delivery [26]. This controlled drug release helps in maintaining localized therapeutic concentrations over extended periods without the need for frequent re-application. Furthermore, the inherent structural stability from covalent bonds and reticular chemistry renders them resistant to degradation and leaching under physiological conditions [25]. The organic nature of CONs ensures better biocompatibility and stability under physiological conditions compared to lipid- or polymeric-based drug delivery nanocarriers. This ensures the protection of therapeutic agents from premature degradation, maintaining efficiency while minimizing potential toxicity [27]. The framework of the CONs can be modified with functional groups or integrated with other biomaterials enabling hybrid formulations for improving targeted capabilities [25,28]. For instance, Zou et al. synthesized a facile one-pot curcumin-loaded COFs incorporated into a polycaprolactone (PCL) nanofibrous membrane to create a pH-responsive drug release platform for wound dressings. They observed a high drug-loading capacity with enhanced mechanical properties, representing a novel approach using COF-based drug-delivery systems for wound dressing applications [15]. Another research group utilized CONs to load and anchor silver nanoparticles (AgNPs), accomplishing antibacterial effects with potential applications as a wound dressing material [29]. The ability to integrate the inherent properties of CONs with drug delivery functions renders significant advantages over conventional systems [30]. 

In this study, the use of CONs as drug delivery carriers was justified by attaching guanidinium units (gCONs) via the Schiff base condensation reaction and loading CUR drug via the wet impregnation method into fabricated guanidinium-covalent organic nanosheets (CUR@gCONs). This study is focused on addressing several key issues: (a) determining the optimal drug loading capacity of gCON; (b) assessing the cytotoxicity of gCON at various concentrations with the human dermal fibroblasts (hDFs); (c) investigating pH-dependent controlled and sustained drug release profiles of CUR; and (d) evaluating the antioxidant and anti-inflammatory potential of CUR. Collectively, our findings underscore the synergistic therapeutic potential of gCON as a stable drug delivery vehicle in conjunction with CUR and address issues like drug bioavailability and instability in the wound microenvironment.

## 2. Experimental Section

### 2.1. Chemicals 

CUR and acetone were purchased from Sigma Aldrich (St. Louis, MO, USA). Guanidinium hydrochloride and dimethylacetamide (DMAc) were purchased from TCI Chemicals (Portland, OR, USA). Hydrazine hydrate was purchased from Alfa Aesar (Ward Hill, MA, USA). Hexamethylenetetramine was purchased from ThermoFisher Scientific (Waltham, MA, USA). Phloroglucinol, dioxane, and dichloromethane were purchased from Acros Organics (Waltham, MA, USA). Trifluoroacetic acid, methanol, and sodium sulfate were purchased from VWR (Radnor, PA, USA). All the chemicals were of analytical grade and used without further purification.

### 2.2. Synthesis Procedure

#### 2.2.1. Synthesis of Guanidinium Covalent Organic Nanosheets (gCON)

**Scheme 1:**
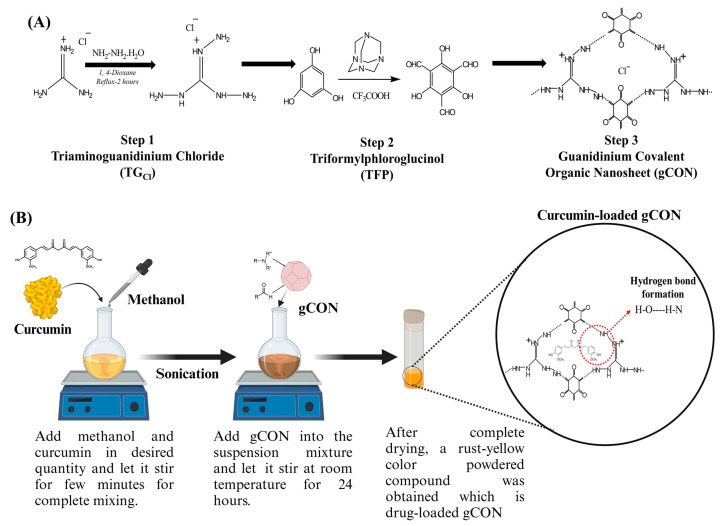
(**A**,**B**): **Diagrammatic representation of the synthesis and incorporation method of CUR onto gCON:** (**A**) Step-by-step synthesis of gCON via the Schiff base condensation method. This schematic outlines the key stages of the reaction process, including reactants, intermediates, and final product, illustrating how gCON is formed through the condensation of amine and aldehyde precursors. (**B**) Incorporation of CUR into gCON via the wet impregnation method. This schematic illustrates the process by which CUR is introduced into the gCON, detailing the steps involved in ensuring effective loading and distribution of CUR within the gCON structure.

The synthesis of gCON is a three-step process, as illustrated in Scheme 1A. 

Synthesis of Triaminoguanidinium Chloride (TG_Cl_): TG_Cl_ was synthesized using hydrazine hydrate in dioxane under reflux reaction conditions. Briefly, guanidinium hydrochloride (1.91 g) was added to dioxane (10 mL) followed by continuous stirring for uniform mixing. Into this suspension, hydrazine hydrate was added (3.41 g). The suspension mixture was stirred under reflux conditions for 2 h. Then, the suspension was cooled at room temperature, followed by dioxane washing to remove excess hydrazine hydrate. The product was dried completely to yield TG_Cl_.

Synthesis of 1,3,5-Triformylpholoroglucinol (TFP): To synthesize TFP, hexamethylenetetramine (7.4 g) and phloroglucinol (3 g) was added all together in trifluoroacetic acid (45 mL) and stirred on ice following the gradual increase in temperature to room temperature. The suspension was further heated at 100 °C for 2.5 h under a nitrogen atmosphere. Then, 3 M hydrochloric acid (HCl) was added dropwise and refluxed for another 1 h with continuous stirring. After this, the suspension was cooled at room temperature and further passed through a Celite (Merck CX0574, Los Angeles, CA, USA) bed. The filtrate obtained was then extracted with dichloromethane (DCM) 4 times and further dried over anhydrous sodium sulfate (Na_2_SO_4_). The extracted solid product was dull yellow and purified via hot ethanol to obtain the desired reaction product. 

Synthesis of Guanidinium Covalent Organic Nanosheets (gCON): The gCON was synthesized through a facile, one-pot Schiff base condensation reaction. The reaction takes place between TG_Cl_ (42 milligrams) and TFP (28 mg) in a sealed tube using a 2:0.6 mL dioxane:water ratio. The mixtures were sonicated for 20 min via a probe sonicator. The reaction mixture was de-gassed three times under liquid nitrogen via freeze-pump thaw cycles following the vacuum sealing of the tube. The reaction is stirred for three days at 120 °C. The gCON (TpTG_Cl_) that was synthesized was brown. The end product was thoroughly washed with dimethylacetamide (DMAc), deionized water, and acetone. The gCON was dried overnight at 70 °C under a vacuum to remove moisture completely. The synthesized gCON has irreversible enol-keto tautomerism with exceptional chemical stability.

#### 2.2.2. Synthesis of Curcumin-Loaded Guanidinium Covalent Organic Nanosheets (CUR@gCON)

The CUR@gCON was synthesized via the wet impregnation method as shown in Scheme 1B. Briefly, the appropriate amount of curcumin was dissolved in 10 mL methanol/0.05 g CUR following probe sonication for 10 min at 25 °C. After complete dissolution, 0.1 g of gCON was added to the CUR solution and further mixed for 24 h at 300–500 RPM and 25 °C. After 24 h of synthesis, the CUR-impregnated gCON was obtained via rotary evaporation at 70 °C, 20–30 RPM for 20 min.

### 2.3. Material Characterization

The textural properties of the gCON were determined via nitrogen (N_2_) isotherms collected on a Micromeritics ASAP 2020 porosimetry analyzer at a temperature of 77.3 K (liquid nitrogen). The Brunauer–Emmett–Teller (BET) surface area of the gCON and 10, 30, and 80 wt% CUR@gCON was determined via the BET technique. Before porosimetry analysis, the samples were degassed under vacuum at 158 °C for 6 h on a Micrometrics Smrt VacPrep system (Norcross, GA, USA). The crystalline property, morphology, functional groups, particle size, and surface charge of pristine gCON, pristine CUR, and CUR@gCON formulations were analyzed via powder X-ray diffraction (PXRD). The spectra were collected on quest ECO Copper source Photon II detector, Billerica, MA, USA), field-emission scanning electron microscope (FE-SEM) (FEI QUANTA 600, Hillsboro, OR, USA), transmission electron microscope (JEOL 1200 EX TEM, Peabody, MA, USA), atomic force microscopy (Bruker Dimension Icon AFM, Billerica, MA, USA), attenuated total reflection Fourier transform infrared (ATR-FTIR) (Bruker ALPHA-Platinum, Billerica, MA, USA) spectra, and particle size analyzer/zeta potential measurements.

### 2.4. Cell Culture Experiments

Human dermal fibroblasts (hDFs) cell lines were procured from ATCC (Manassas, VA, USA). hDFs were cultured in Dulbecco’s modified Eagle Medium (DMEM) (ThermoFisher Scientific) supplemented with 20% fetal bovine serum (FBS) (R&D Systems, Atlanta, GA, USA), 20% Ham F12 (Life Technologies Corporation, Carlsbad, CA, USA), and 1% penicillin/streptomycin (Life Technologies Corporation). Cells were cultured in 75 cm^2^ tissue culture flasks at 37 °C in the incubator with 5% supplied carbon dioxide (CO_2_). For proper maintenance of the cells, the culture media was changed once every three days. 

#### Live/Dead Viability Assay

The biocompatibility of the gCON was assessed via the Invitrogen™ LIVE/DEAD^®^ Viability/Cytotoxicity Assay Kit(Waltham, MA, USA). Briefly, 0.1 million hDF cells were seeded in a 24-well plate. After overnight incubation, the cells were exposed to different concentrations of gCON (100, 250, 500, and 1000 µg/mL) for two different time points, 48 h and 72 h. The experiment was performed in triplicate (*n* = 3). At the appropriate time point, the cells were washed with Dulbecco’s Phosphate-Buffered Saline (D-PBS). Add 100–150 µL working solution of combined LIVE/DEAD^®^ assay reagents (Calcein AM and Ethidium Homodimer-1, Waltham, MA, USA) in the well-containing cells and samples. Incubate the treated cells for 30–45 min at room temperature. View the labeled treated cells under the fluorescence microscope and images were captured at 10× magnification.

### 2.5. Drug Release Studies

Phosphate-Buffered Saline (PBS) was considered an analog for the human body from the standpoint of drug delivery experiments. Briefly, twenty milligrams of CUR-loaded gCON (80 wt%) were dissolved in 10 mL PBS containing 10% ethanol at different pH values, that is 5.0 and 7.5, respectively. The suspension solution was stirred continuously at 37 °C for different time intervals, 24 h, 48 h, 72 h, 96 h, and 120 h. At pre-determined time points, 2 mL of release medium was taken out for testing the release profile and further replaced with the same amount of fresh media solution. The withdrawn release medium was centrifuged for 15 min at 6000–8000 RPM. The supernatant was withdrawn and evaluated via plate reader (BioTek Cytation 5, Winooksi, VT, USA) by measuring absorbance at 427 nm. The percentage release of the curcumin was calculated from the equation:

$$\mathrm{Percentage}\;(\%)\;\mathrm{Drug}\;\mathrm{Release}=\frac{\mathrm{Mt}}{M0}\;\times \;100\%$$

where Mt is the total amount of CUR released at time t and M0 is the amount of CUR loaded in the gCON. The experiment was performed in triplicate (*n* = 3).

### 2.6. Curcumin-Loaded gCON Bioactivities

#### 2.6.1. DPPH (2,2-Diphenyl-1-Picrylhydrazyl) Free Radical Scavenging Assay

The antioxidant activity of CUR-loaded gCON was assessed through a DPPH assay. Briefly, 1 g CUR@gCON (80%), ascorbic acid (positive control), pristine gCON (negative control), and ethanol (control) were prepared at different concentrations via serial dilution. The mixture was dissolved by stirring them for 2–3 h at 500–700 RPM at room temperature. Next, the samples were centrifuged for 15 min at 6000–8000 RPM following the filtration of supernatant using Whatman filter paper. This is termed an extracted solution. DPPH was weighed and dissolved in absolute ethanol to make a 1 M solution. Following this, 1 mL of each sample was mixed with 3 mL of DPPH solution (1 M). The final volume was adjusted to 10 mL by adding absolute ethanol. The samples were incubated for 30 min in the dark. After incubation, the absorbance was measured at 517 nm using a plate reader. The experiment was performed in triplicate.

#### 2.6.2. Nitric Oxide (NO) Assay

THP-1 monocytes procured from ATCC were cultured with RPMI-1640 media supplemented with 10% FBS (R&D systems) and 1% penicillin/streptomycin (ThermoFisher Scientific). THP-1 monocytes were treated with freshly prepared Phorbol 12-myristate 13-acetate (PMA, Sigma Aldrich) (50 ng/mL) supplemented RPMI-1640 media for 24 h to promote their differentiation into a macrophage. After 24 h of PMA stimulation, almost all THP-1 cells become adherent. THP-1-derived macrophages were trypsinized and seeded in a 96-well plate with a seeding density of 20,000 cells/well. After seeding, the macrophages were treated with pristine gCON, pristine CUR, and different concentrations of CUR@gCON (80%), that is 100, 250, 500, and 1000 µg/mL for 48 h and 72 h, respectively. To analyze the NO scavenging activity, Griess Reagent (Invitrogen™ G7921, ThermoFisher Scientific) was used to measure the nitrite amount, which is a metabolite of nitric oxide. The Griess assay is a two-step diazotization reaction. Briefly, sulfanilic acid is converted quantitatively into a diazonium salt when reacted with the nitrite present in the acid solution. Furthermore, the generated diazonium salt is combined with N-(1-naphthyl) ethylenediamine dihydrochloride, to form a chromophoric azo dye which is detected spectrophotometrically. We followed the manufacturer’s guidelines to perform the assay. The experiment was performed in triplicate (*n* = 3). The absorbance of the samples was measured at 548 nm and the percentage of nitric oxide inhibition was calculated from the equation:

$$\mathrm{Percentage}\;(\%)\;\mathrm{of}\;\mathrm{NO}\;\mathrm{radical}\;\mathrm{scavenging}\;=\;\frac{\left(A_{0}-A_{1}\right)}{A_{0}}\times 100$$

where *A*_0_ stands for absorbance of the control and *A*_1_ stands for absorbance of the treated samples [31,32]. 

### 2.7. Statistical Analysis

The experiments were performed in triplicates (*n* = 3) and the data are presented as mean ± SD. The analysis was carried out using a two-way analysis of variance (ANOVA) and Tukey’s post hoc tests using GraphPad Prism software (Version 8). Results were considered statistically significant for * *p* < 0.05, ** *p* < 0.01, *** *p* < 0.001, **** *p* < 0.0001.

## 3. Results and Discussion

### 3.1. Synthesis and Characterization of gCON

**Figure 1:**
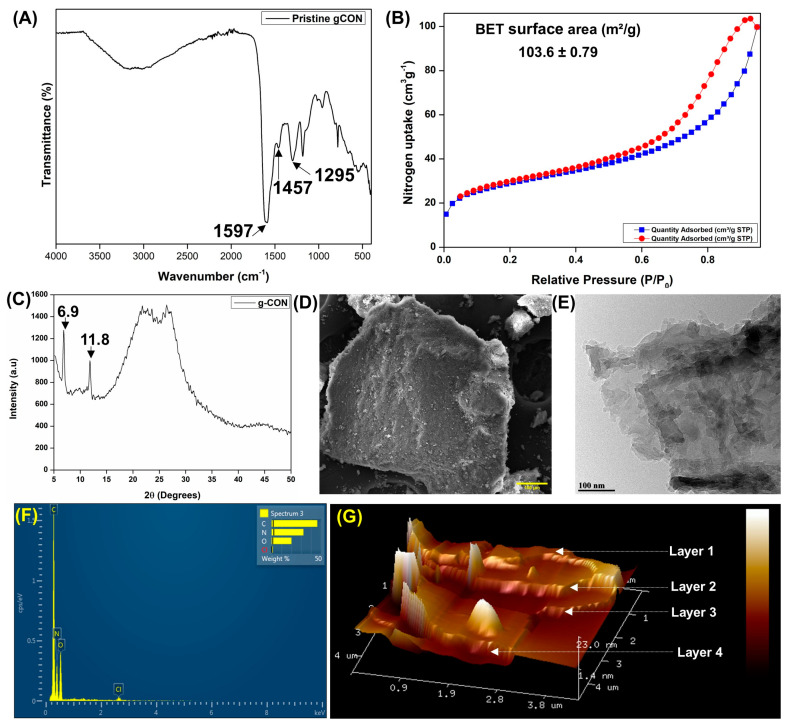
(**A**–**G**): **Material characterization of pristine gCON:** (**A**) FTIR Spectra depicting characteristic functional groups on the surface of gCON, confirming its chemical composition and successful synthesis. (**B**) N_2_ adsorption isotherm analyzed via BET determining gCON’s porosity. (**C**) PXRD pattern displays the crystallinity of the gCON. (**D**,**E**) FE-SEM (scale bar: 100 μm) and TEM (scale bar: 100 nm) exhibit gCON’s marginally rippled sheet-like surface topography. (**F**) EDS for compositional analysis, revealing the elemental distribution of gCON. (**G**) A 3D AFM image depicting the multi-layer stacked morphology of self-exfoliated gCON layers.

The gCON synthesis is a one-pot procedure, in which a Schiff base condensation reaction occurs between TFP and TG_Cl_. Guanidinium units were incorporated into the covalent organic nanosheet framework by synthesizing TG_Cl_, followed by the self-exfoliation of the framework through interlayer repulsion. The ATR–FTIR spectrum of gCON exhibited characteristic peaks, providing crucial information regarding functional groups. Specifically, C=N stretching vibrations at 1593 cm^−1^ are a hallmark of the Schiff base formation, integral to gCON synthesis [33]. Additionally, C-C and C-O stretching vibrations at 1289 cm^−1^, suggest the presence of carbon-carbon and carbon-oxygen bonds in the gCON structure, rendering structure and stability. The peak at 1466 cm^−1^ signifies the antisymmetric vibrations of -CH_3_, suggesting the presence of methyl groups in the gCON, which also contributes to the stability of gCON (Figure 1A) [34,35]. Altogether, these spectral features confirm the gCON synthesis.

The porosity of the gCONs was examined via N_2_ adsorption measurements of the activated sample at 77 K. gCON with BET surface area of 103.6 ± 0.79 m^2^/g exhibited a Type-IV reversible adsorption isotherm, indicating multilayer capillary condensation typically observed in mesoporous materials [36] (Figure 1B). To determine the crystalline nature of gCON, PXRD analysis was performed. The PXRD pattern revealed low crystallinity, with sharp peaks at 2θ = 6.9 and 2θ = 11.8° (Figure 1C). The broad peak suggests poor π-π stacking between vertically stacked nanosheet layers, indicating the presence of loosely bound chloride (Cl^−^) ions and positively charged guanidinium ions, further confirming the low crystallinity of the gCON. 

The morphology of gCON was observed using FE-SEM (Figure 1D) and TEM (Figure 1E). FE-SEM revealed that the self-exfoliation of gCON, driven by the guanidinium units, led to the formation of a sheet-like structure. EDS analysis revealed the presence of carbon (C), nitrogen (N), oxygen (O), and chloride ions (Cl) as the main components of gCON (Figure 1F). The TEM images indicated the formation of thin transparent sheets that were marginally rippled, aligning with the findings reported in the literature [34,35]. The thickness of the gCON was determined by AFM, which showed a few layers of the self-exfoliated sheets with a measured height of 2–7 nm, indicating self-exfoliation into 3–4 layers (Figure 1G). 

### 3.2. Cytocompatibility Testing

**Figure 2:**
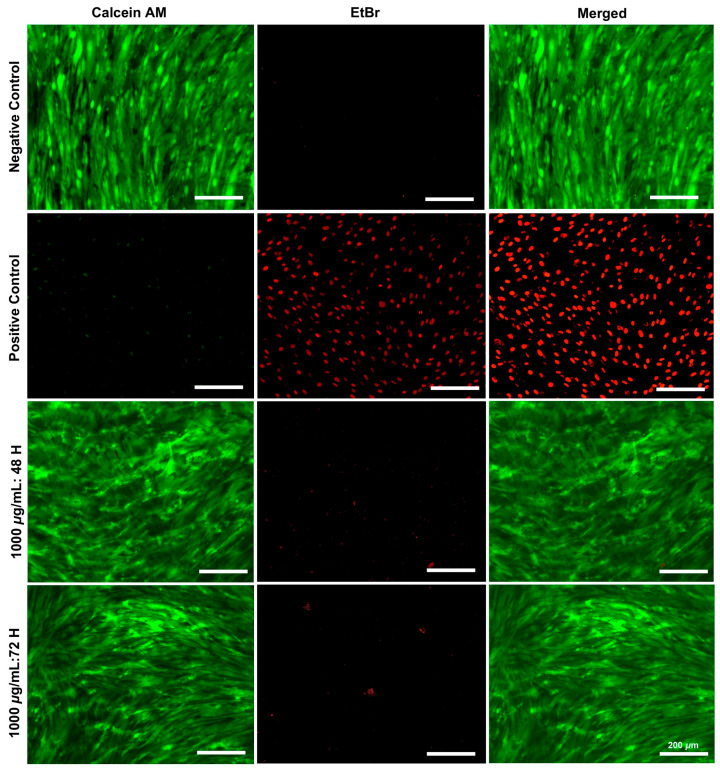
**Cytocompatibility testing of pristine gCON:** Live/Dead assay of hDFs exposed to 1000 µg/mL of pristine gCON for 48 h and 72 h. Fewer dead cells (red fluorescence) and increased cell density (green fluorescence) at 72 h indicating low toxicity and biocompatibility even at higher concentrations. DMSO-treated cells serve as positive control. Scale bar: 200 μm.

The LIVE/DEAD assay indicated good cytocompatibility of gCON at different concentrations for 48 h and 72 h, respectively (Figure 2 and Figure S1A,B). After 48 and 72 h of cell seeding, minimal to no dead cells (red fluorescence) were observed, along with an increased cell density indicating live cells (green fluorescence) compared to the positive and negative controls. A significant increase in cell density was observed even at the highest concentration of 1000 µg/mL (Figure 2). This can be attributed to the surface charge of the gCON impacting its cell internalization [37] and the formation of the protein corona-a protective layer [38]. The positively charged guanidinium units are readily taken up by cells due to the negatively charged phospholipid bilayer of the cell membranes, leading to electrostatic interactions [29,39] through numerous endocytosis pathways like micropinocytosis, clathrin-mediated endocytosis, and caveolae-mediated endocytosis, and supports cellular survival and metabolic activity [37,40]. These electrostatic forces play a pivotal role in how nanoparticles interact with cells, including how they form protein corona and affect cellular viability [41]. 

As discussed by Walkey et al. [42], when a positively charged gCON enters a biological environment, such as cell culture media, negatively charged proteins like albumin and fibrinogen from the surrounding can easily adsorb onto its surface forming protein corona. At higher concentrations, more positively charged gCON particles are available, each with a larger surface area to interact with biomolecules [43,44]. This leads to greater protein adsorption, forming a richer and more stable protein corona. The protein corona acts as a bridge between nanoparticles and cells, enhancing cellular attachment and proliferation by mimicking ECM proteins and activating integrin-mediated signaling pathways. In contrast, at lower concentrations, fewer nanoparticles are present, resulting in a limited surface area and incomplete protein corona formation. This exposes the cytotoxic sites of the bare nanoparticle surface, potentially causing oxidative stress, disrupting membrane integrity, and interfering with cellular functions [45]. Overall, pristine gCON showed less toxicity and supported the adhesion and proliferation of hDFs, indicating its potential as a biocompatible drug delivery system for wound healing applications.

### 3.3. Material Characterization and Bioactivity Testing of CUR@gCON Formulations

**Figure 3:**
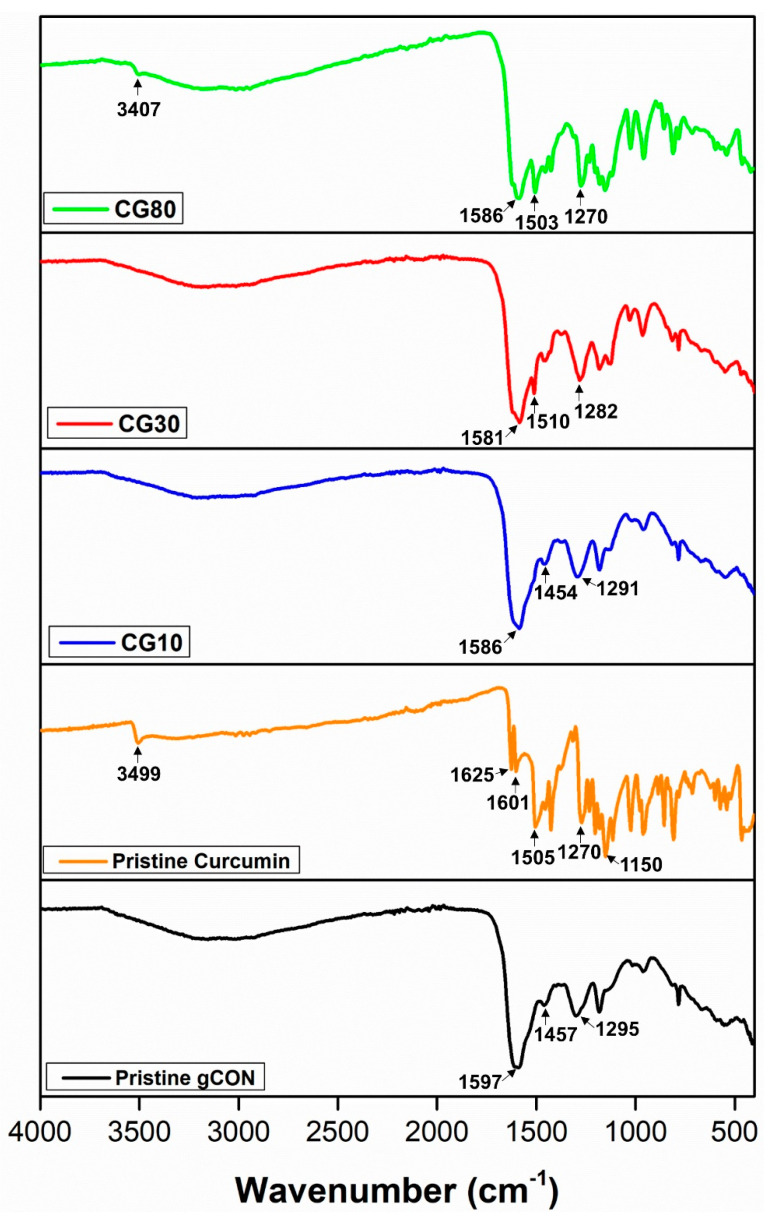
**FTIR spectra of pristine CUR, pristine gCON, and CUR@gCON formulations.** The spectrum confirms the successful loading of CUR into gCON. The distinct peaks for each component and CUR@gCON formulations indicate the interactions between CUR and gCON, verifying effective loading.

As confirmed by porosimetry analysis, the porous gCONs are good hosts for loading functional guest molecules. CUR (C_21_H_20_O_6_; MW = 368.38 g/mol) was loaded in gCON in three CUR@gCON formulations: 10, 30, and 80 wt%. All three formulations of CUR@gCON exhibited similar FTIR spectra (Figure 3). In pristine CUR, the peak at 3499 cm^−1^ signifies O-H stretching due to hydroxyl groups, 1601 and 1625 cm^−1^ signifies C=O stretching, indicating the presence of keto carbonyl groups, 1505 cm^−1^ indicates aromatic C=C stretching, and 1270 cm^−1^ signifies C-O stretching, which is related to the stretching of C-O bond in the phenolic group. In all CUR@gCON formulations, the peaks around 1500 cm^−1^ signify C=N stretching vibrations, while the peak around 1291 cm^−1^ signifies C-N stretching in the gCON and C-O stretching in the curcumin. The peak around 3400 cm^−1^ signifies free O-H stretching. These results indicate that the impregnation of CUR at different drug concentrations occurs due to hydrogen bond formation between CUR and gCON. This also shows that drug loading did not induce any changes in the bond structures of gCON. Therefore, the CUR-loading mechanism suggested complete physical drug impregnation and did not originate from any type of chemical reaction [46]. 

**Figure 4:**
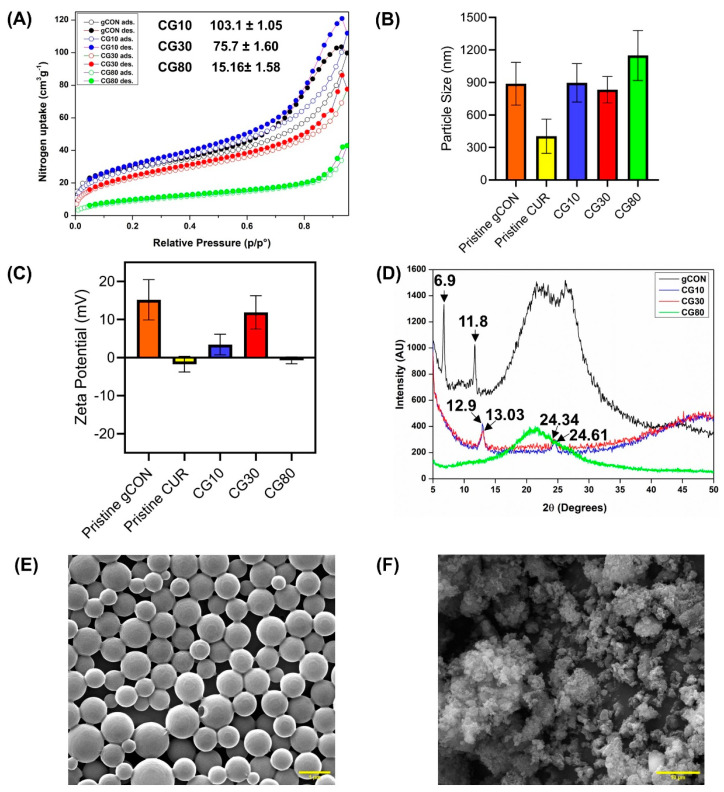
(**A**–**F**): **Material characterization pristine CUR and CUR@gCON formulations:** (**A**) N_2_ adsorption isotherm via BET showing almost complete filling of gCON pores after CUR loading, indicating successful incorporation; (**B**) Particle size analysis indicating increased hydrodynamic diameter incorporation of CUR into the gCON; (**C**) Zeta potential measurements reveal the near-neutral surface charge of CUR@gCON due to charge neutralization effects; (**D**) PXRD spectra illustrating changes in the crystalline structures upon CUR loading onto gCON; (**E**) FE-SEM image of pristine CUR shows spherical morphology with an average diameter of 17 μm. Scale bar: 1 μm, and (**F**) FE-SEM image of CUR@gCON (80%) displays altered gCON topography due to pore saturation and CUR loading, highlighting differences in structure, post-loading. Scale bar: 10 μm.

The N_2_ physisorption isotherms showed that the surface area of the gCON decreased as the CUR loading increased from 10% to 80%, ultimately filling the pores of gCON (Figure 4A). This indicated that high CUR loading led to complete saturation of the pristine gCON surface, governed by the near-complete exhaustion of gCON’s N_2_ physisorption capacity. The hydrodynamic diameters of pristine gCON and pristine CUR were 896.9 ± 197.3 nm and 404.0 ± 58.34 nm, respectively. The hydrodynamic diameter of the CUR@gCON formulations increased to 888.8 ± 77.81 nm (10%), 833.5 ± 121.5 nm (30%), and 1149 ± 230.7 (80%), indicating successful incorporation of CUR into the gCON skeleton (Figure 4B). The surface charge of pristine gCON was +15.2 ± 0.289 mV attributed to the guanidinium cations, whereas the surface charge of the pristine CUR was −1.71 ± 2.07 mV, respectively (Figure 4C). The zeta potential of CUR@gCON formulations was decreased to 3.45 ± 0.711 mV (10%), 11.9 ± 2.39 mV (30%), and −0.7 ± 0.884 mV (80%), respectively. In the case of CUR@gCON 80%, the slightly negative but close to zero zeta potential value was observed due to the neutralizing effect of opposite charges of gCON and CUR (Figure 4C). 

The PXRD analysis of CUR@gCON (Figure 4D) signified the completely amorphous nature of the material with the elimination of the two peaks at degrees 6.9 and 11.8. The presence of peaks in CG10 and CG30 at 12.9°, 13.03°, 24.34°, and 26.61° are the characteristic peaks of the CUR crystal lattice. At lower drug loadings (10% and 30%), there was intermolecular interaction between CUR and gCON pores, whereas at high drug loading (80%), electrostatic interactions were also observed between the oxygen atom of the positively charged gCON and the hydrogen atom of the negatively charged CUR. At 80% drug loading, the gCON pores were completely saturated, and additional CUR molecules were deposited on the surface of the gCON, observed as a broad hump due to strong hydrogen bonding occurring between the individual CUR molecules. A higher surface area of gCONs can result in higher loading of the drug without any hindrance to diffusion pathways during drug delivery [19]. Furthermore, the FE-SEM analysis of the pristine CUR revealed spherical morphology with an average diameter of 17 μm. In contrast, the CUR@gCON formulations (Figure 4E,F) demonstrated the destruction of the topographical characteristic features of the pristine gCON, depicting the saturation of gCON pores with an absence of crystal faces via drug loading and coating the gCON surface.

**Figure 5:**
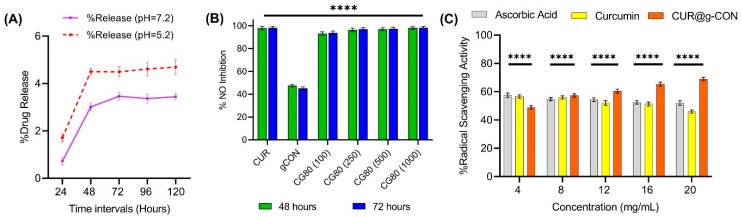
(**A**–**C**): **Bioactivity testing of pristine gCON, pristine CUR, and CUR@gCON formulation:** (**A**) Release rate profiles of CUR from CUR@gCON (80%) at two different pH levels, 7.2 and 5.0, illustrating the pH-dependent release behavior of CUR in different biological environments; (**B**) Antioxidant activity assessment via DPPH assay comparing the relative scavenging effects of each formulation in neutralizing free radicals; and (**C**) Anti-inflammatory activity evaluation via the Griess reagent reaction, measuring nitric oxide production as a marker of inflammatory response. CUR@gCON exhibits potent anti-inflammatory properties compared to pristine gCON (**** *p* < 0.0001).

To evaluate the characteristic properties of gCON for drug delivery, the percentage of CUR release from CUR@gCON (80%) formulation was determined (Figure 5A). Approximately 5% (pH 5.2) and 3% (pH 7.2) of CUR were released over 120 h. Lawson et al. encapsulated CUR in MOF [46], resulting in a significantly reduced drug release. They attributed this phenomenon to high drug loading, which blocks the diffusive pathways within the pores and enhances pharmacokinetics by slowing the surface drug release rate. Similarly, our higher CUR loading in gCON may have acted as a protective barrier, contributing to the overall slow cumulative drug release over 120 h. In addition, the imine groups in CUR@gCON significantly increased the number of conjugated nitrogen (N) atoms in the drug-loaded nanosheet, which resulted in pH-dependent CUR release behavior. Furthermore, the charge attraction between curcumin and gCON played a crucial role in the sustained release of CUR. Therefore, the CUR@gCON drug delivery system demonstrates the pH-responsive CUR release when it encounters a target, such as a wound bed. 

The anti-inflammatory properties of the CUR@gCON were investigated in vitro using the THP-1 macrophage-like cell model. Notably, the highest concentration of CUR@gCON (1000 µg/mL) demonstrated nearly 98% NO free radical scavenging, compared to the pristine curcumin (~97%) and pristine gCON (~47%) at 48 and 72 h, respectively (*p* < 0.0001) (Figure 5B). As aforementioned, 80% of CUR loading fully saturated the gCON pores, leaving additional CUR molecules on the gCON surface. These free CUR molecules likely contributed to the enhanced NO inhibition observed when CUR@gCON was cultured with THP-1 macrophage-like cells and analyzed via the Griess assay. Thus, CUR@gCON exhibits promising anti-inflammatory properties, particularly in addressing the stalled inflammatory phase of wound healing. 

To evaluate the antioxidant bioactive property of CUR@gCON, a DPPH free radical scavenging assay was performed at concentrations ranging from 4 to 20 mg/mL, using ascorbic acid as a positive control. The assay demonstrated the free radical scavenging activity of CUR and CUR@gCON (Figure 5C); CUR has a natural antioxidant activity and its low availability limits its radical scavenging effectiveness. CUR@gCON showed significantly higher radical scavenging activity at higher concentrations at 20 mg/mL (69 ± 0.23%) compared to the pristine CUR (45 ± 0.07%) (*p* < 0.0001). Ascorbic acid showed a significant reduction in antioxidant activity, decreasing from 57 ± 0.6% to 52 ± 0.5% (*p* < 0.0001) as the concentration increased from 4 to 20 mg/mL. The encapsulation of CUR in gCON enhances its stability by forming a stable matrix around CUR molecules due to strong covalent bonds in the gCON framework. This protective matrix shields CUR from environmental degradation factors, preventing premature breakdown [27]. Furthermore, as a hydrophobic drug, CUR typically has poor solubility in aqueous environments; however, gCON encapsulation prevents the formation of large, insoluble CUR aggregates [47]. gCON renders a hydrophilic environment improving the solubility and bioavailability, enabling therapeutic levels over extended periods and better interaction with DPPH radicals [48]. These results indicate that the compounds can donate hydrogen (H) atoms to neutralize free radicals, explaining the observed antioxidant activity. gCON not only served as a nanocarrier but also as a ROS scavenging agent, further improving curcumin’s antioxidant activity. This synergistic structural enhancement resulted in better scavenging performance in the DPPH assay, surpassing that of ascorbic acid [49]. Therefore, CUR@gCON overcomes the bioavailability limitations of pristine CUR, showing significantly enhanced antioxidant properties. 

## 4. Conclusions

A 2D curcumin-loaded guanidinium covalent organic nanosheets (CUR@gCONs) was successfully synthesized using the Schiff base condensation reaction and wet impregnation method. The filling of gCON pores via CUR was confirmed through BET surface area. The morphological investigations revealed the formation of sheets in gCON, with CUR loading causing noticeable disruption of sheet-like structure. When cultured with hDFs, gCON demonstrated cytocompatibility even at higher concentrations. As evidenced by the drug release rate studies, gCON facilitated the sustained release of the CUR over an extended period of 5 days, thereby enhancing its therapeutic potential. Furthermore, the inherent antioxidant and anti-inflammatory properties of CUR were preserved after loading into the gCON delivery carrier. Conclusively, this highlights the synergistic behavior of CUR@gCON, combining gCON’s efficacy as a nanocarrier with curcumin’s pleiotropic effects for potentially improving wound healing via efficient drug delivery. 

## Data Availability

The data presented in this study are available on request form the corresponding author.

## Supplementary Materials

The following supporting information can be downloaded at: https://www.mdpi.com/article/10.3390/biomimetics9110709/s1, Figure S1: (A,B): Cytocompatibility testing of pristine gCON: Live/Dead viability assay of pristine gCON exposed at different concentrations (100, 250, 500, and 1000 µg/mL) on hDFs for (A) 48 h and (B) 72 h, respectively. Fewer dead cells (red fluorescence) and increased cell density (green fluorescence) indicating low toxicity and cytocompatibility even at higher concentrations. DMSO-treated cells serve as positive control. Scale bar: 200 μm.
