# Supplementary material for: Self-Exfoliated Guanidinium Covalent Organic Nanosheets as High-Capacity Curcumin Carrier
Source: Biomimetics (Basel). 2024 Nov 19;9(11):709. doi: 10.3390/biomimetics9110709 (PMC11592196; doi:10.3390/biomimetics9110709)
Supplement: Supplementary file 1 [file biomimetics-09-00709-s001.zip › biomimetics-3299089-supplementary.pdf]

Supporting Information

Self-Exfoliated Guanidinium Covalent Organic Nanosheets as High-Capacity Curcumin Carrier

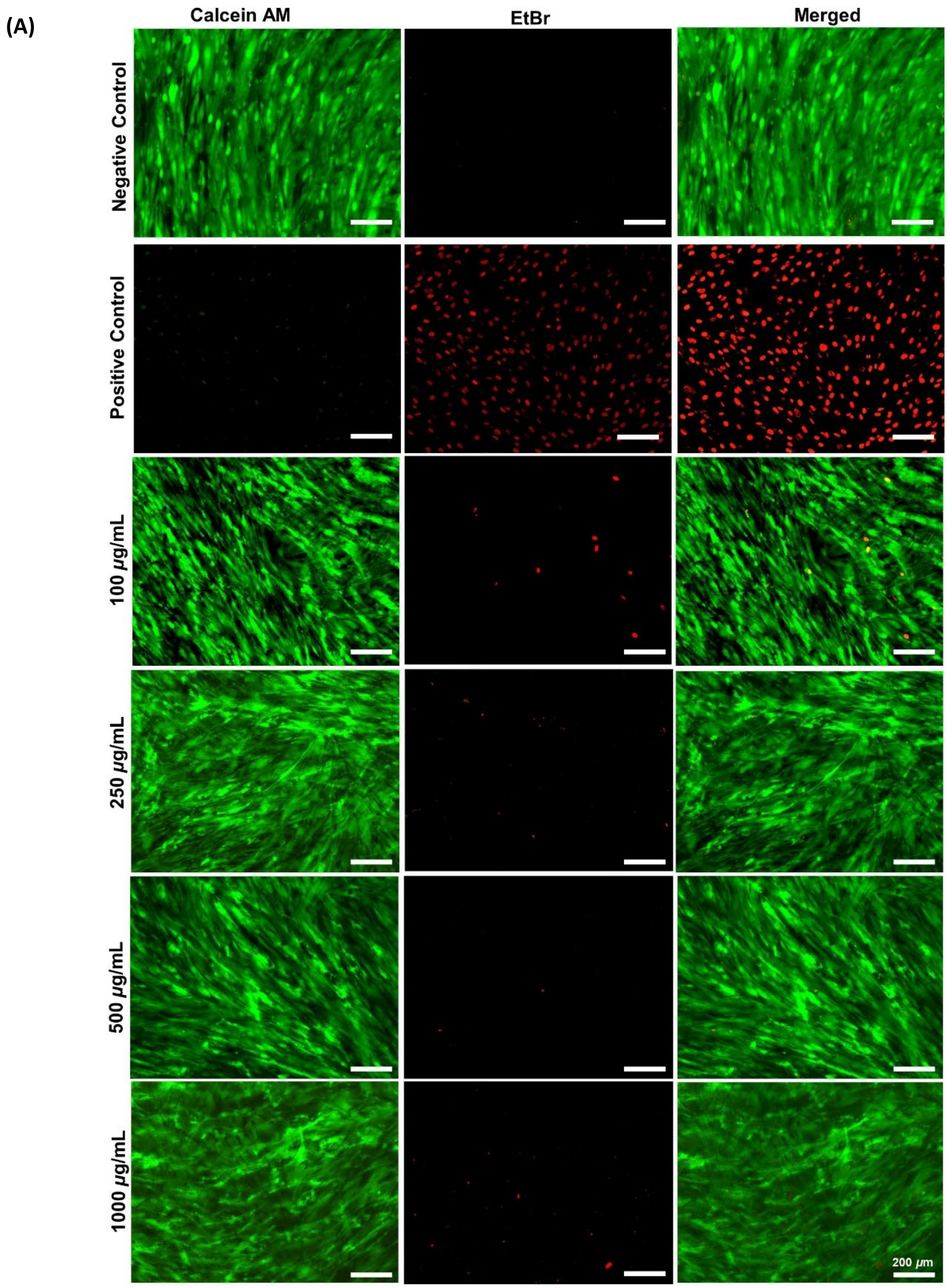

(A)
Calcein AM
EtBr
Merged
Negative Control
Positive Control
100 μg/mL
250 μg/mL
500 μg/mL
1000 μg/mL
200 μm

(B)

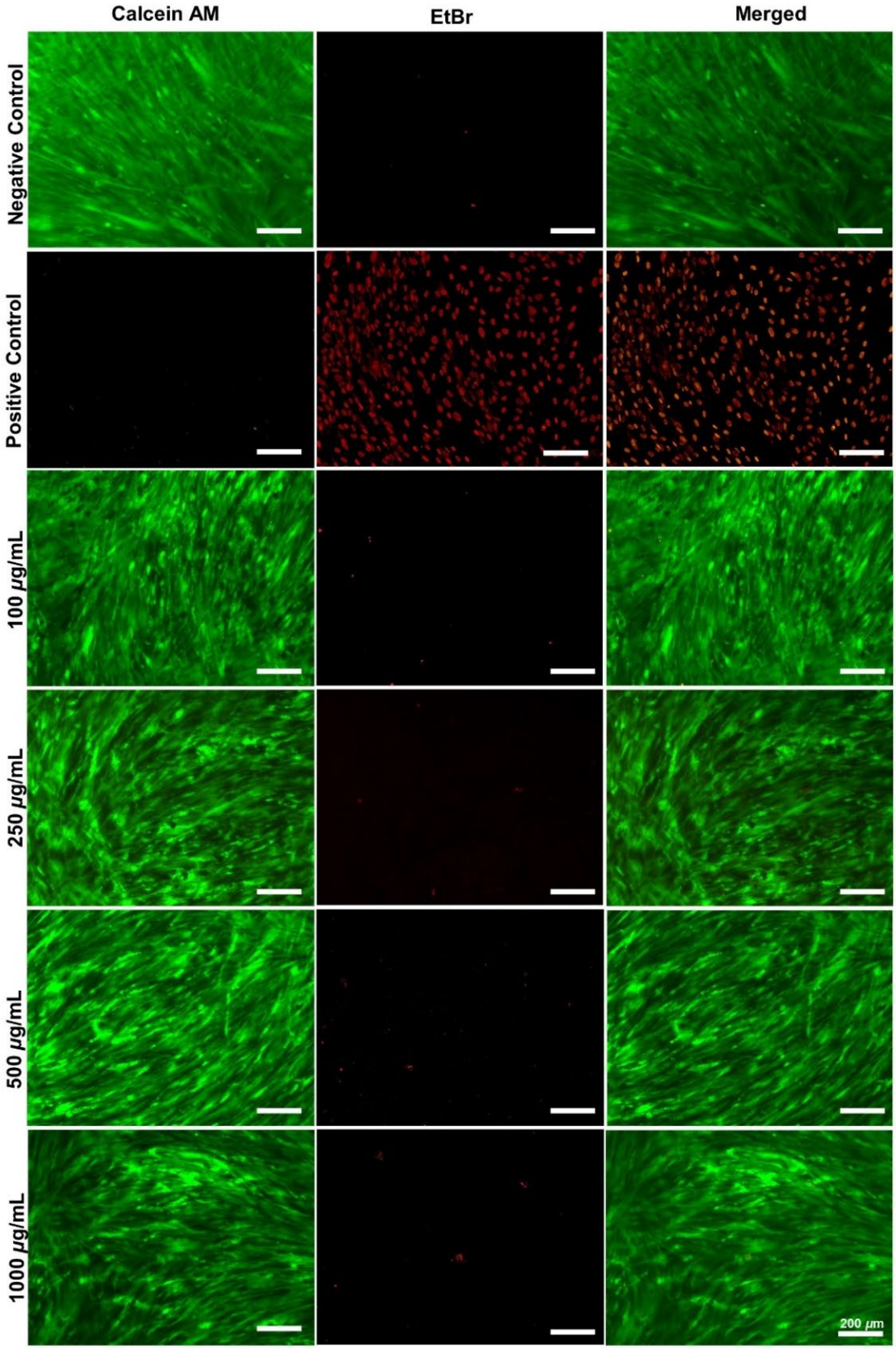

Figure S1 (A-B): Cytocompatibility testing of pristine gCON: Live/Dead viability assay of pristine gCON exposed at different concentrations (100, 250, 500, and 1000 μg/mL) on hDFs for **(A)** 48 hour and **(B)** 72 hours, respectively. Fewer dead cells (red fluorescence) and increased cell density (green fluorescence) indicating low toxicity and cytocompatibility even at higher concentrations. DMSO-treated cells serve as positive control. Scale bar: 200 μm.
